# Nutritional Status Is Associated with Bone Mineral Density, Vitamin D Levels, and Bone Turnover Markers in Patients with Proximal Femoral Fragility Fractures: A Retrospective Observational Study

**DOI:** 10.3390/medicina62061092

**Published:** 2026-06-04

**Authors:** Masahiro Iinuma, Takahiro Hideshima, Shinji Machida, Kenji Uehara, Tomoko Karube, Kentaro Sato, Naoki Haraguchi

**Affiliations:** 1 Department of Orthopaedic Surgery, St. Marianna University Yokohama Seibu Hospital, Yokohama 241-0811, Kanagawa, Japan; 2 Department of Orthopaedic Surgery, St. Marianna University School of Medicine, Kawasaki 216-8511, Kanagawa, Japan; 3 Department of Nephrology, St. Marianna University Yokohama Seibu Hospital, Yokohama 241-8511, Kanagawa, Japan

**Keywords:** fragility fracture, hip fracture, nutritional status, bone mineral density, bone turnover markers, GNRI

## Abstract

*Background and Objectives*: Malnutrition is common among older adults with fragility fractures and is linked to poor clinical outcomes in orthopedic surgery. However, the association between nutritional status and bone-related parameters, including bone mineral density (BMD) and bone turnover markers, remains inadequately characterized in this population. This study evaluated these associations in patients with proximal femoral fragility fractures. *Materials and Methods*: In total, 108 patients who underwent surgery for proximal femoral fragility fractures were retrospectively analyzed. Nutritional status was evaluated using the Geriatric Nutritional Risk Index (GNRI), Prognostic Nutritional Index (PNI), and Controlling Nutritional Status (CONUT) score. BMD was measured at the femoral neck of the proximal femur and the lumbar spine using dual-energy X-ray absorptiometry. Serum 25-hydroxyvitamin D (25[OH]D) and bone turnover markers, including total procollagen type I N-terminal propeptide and tartrate-resistant acid phosphatase 5b, were also evaluated. Correlation analyses, group comparisons, and multivariate linear regression analysis were performed to identify factors independently associated with femoral neck BMD. *Results*: GNRI and PNI were significantly positively correlated with femoral neck BMD (r = 0.48 and r = 0.26, respectively; both *p* < 0.01), while the CONUT score showed a significant negative correlation (r = −0.27, *p* < 0.01). Nutritional indices were not significantly correlated with lumbar spine BMD. Patients classified as malnourished by GNRI or PNI had significantly lower femoral neck BMD and serum 25(OH)D and higher bone turnover markers than well-nourished patients. Multivariate linear regression analysis revealed that GNRI, PNI, and CONUT score remained independently associated with femoral neck BMD after adjusting for age, sex, and body mass index. *Conclusions*: Nutritional status assessed by hematological indices was significantly associated with femoral neck BMD and bone metabolism markers in patients with proximal femoral fragility fractures. Findings underscore the importance of nutritional status in bone health and should be considered in the management of osteoporosis and fragility fractures.

## 1. Introduction

The number of older patients requiring orthopedic treatment following fragility fractures has steadily increased, with the rapid aging of populations. Patients with fragility fractures are often characterized by advanced age, frailty, and multiple comorbidities and are thus at high risk for perioperative complications. Accumulating evidence in the field of orthopedic surgery has demonstrated the association of malnutrition with postoperative complications, prolonged hospital stay, impaired functional recovery, and higher mortality. Accordingly, nutritional status has emerged as a key determinant of perioperative risk [1,2]. In particular, patients with fragility fractures frequently exhibit a complex pathological interplay among malnutrition, inflammation, and frailty. Studies focusing on older adults with fractures, along with international consensus statements, have consistently emphasized the importance of appropriate nutritional assessment in this population [3,4].

Practical nutritional screening indices currently used in clinical practice include the Geriatric Nutritional Risk Index (GNRI) [5], the Prognostic Nutritional Index (PNI) [6], and the Controlling Nutritional Status (CONUT) score [7]. These indices are based on routinely available laboratory and anthropometric parameters, facilitating the objective assessment of nutritional risk.

Malnutrition is a risk factor for osteoporosis and may increase fracture risk by disrupting bone metabolism and reducing bone mass [8,9]. Although an association between nutritional status and bone mineral density (BMD) has been reported [10,11], comprehensive investigations of these relationships in orthopedic surgery remain limited, particularly among patients with proximal femoral fragility fractures. Therefore, the present study elucidated the association of nutritional status, assessed using hematological indices, with BMD and bone turnover markers in patients with proximal femoral fragility fractures.

## 2. Materials and Methods

### 2.1. Study Population

This single-center retrospective observational study included patients who underwent surgery for proximal femoral fragility fractures between April 2022 and December 2025. The inclusion criteria were as follows: patients aged ≥ 60 years who underwent surgery for proximal femoral fragility fractures and had available data on nutritional indices, BMD, vitamin D levels, and bone turnover markers. The exclusion criteria were as follows: patients aged < 60 years and those with missing data required for the analysis, including height, body weight, laboratory data required to calculate nutritional indices, BMD measurements, serum 25(OH)D levels, or bone turnover markers. Patients aged < 60 years were excluded because younger patients may include those with high-energy trauma or clinical backgrounds different from those of older patients with fragility fractures. The study was approved by the Institutional Review Board, and written informed consent was obtained from all participants. The initial screening included 216 patients. Of these, 102 patients with missing data and 6 patients aged < 60 years were excluded, resulting in a final cohort of 108 patients.

### 2.2. Data Collection and Study Variables

The study variables analyzed in this study included patient characteristics (age, sex, body mass index [BMI], diagnosis, and surgical procedure); nutritional indices (GNRI, PNI, and CONUT), calculated from blood tests obtained 5.2 ± 3.9 days before surgery (mean ± SD); BMD assessed by dual-energy X-ray absorptiometry (DXA) at 7.9 ± 5.3 days after surgery; laboratory findings; and pre-fracture osteoporosis treatment status.

At our institution, patients with proximal femoral fragility fractures are routinely evaluated for osteoporosis and secondary fracture prevention. As part of this institutional protocol, BMD, serum 25(OH)D levels, and bone turnover markers are generally measured to guide postoperative osteoporosis treatment.

Femoral BMD was evaluated at the femoral neck. Lumbar spine BMD was defined as the mean T-score of L1–L4. Vertebrae with compression fractures or marked degenerative changes were excluded. Furthermore, the mean T-score was calculated using only evaluable vertebrae. The laboratory findings assessed included corrected calcium, phosphorus, albumin, estimated glomerular filtration rate, and C-reactive protein, which were measured 5.2 ± 3.9 days preoperatively. Serum 25-hydroxyvitamin D (25[OH]D) and bone turnover markers, including total procollagen type I N-terminal propeptide (total P1NP) and tartrate-resistant acid phosphatase 5b (TRACP-5b), were measured 3.5 ± 5.7 days postoperatively. Measurements of 25(OH)D, total P1NP, and TRACP-5b were outsourced to LSI Medience Corporation (Tokyo, Japan). Serum 25(OH)D was measured using an electrochemiluminescence immunoassay (ECLIA) with the Elecsys Vitamin D Total III assay (Roche Diagnostics K.K., Tokyo, Japan). Total P1NP was measured using ECLIA with the Elecsys total P1NP assay (Roche Diagnostics K.K.). TRACP-5b was measured using an enzyme immunoassay (EIA) with the N-Test EIA Plate TRACP-5b kit (Nittobo Medical Co., Ltd., Tokyo, Japan).

### 2.3. Nutritional Assessment

In this study, nutritional status was assessed using three hematological nutritional indices: the GNRI, PNI, and CONUT score. The GNRI was developed by Bouillanne et al. [5] in 2005 to assess nutritional risk specifically in older populations. It is calculated using the following formula: GNRI = (14.89 × serum albumin [g/dL]) + (41.7 × actual body weight/ideal body weight). Nutritional status is stratified into four categories based on the calculated score: no risk (>98), low risk (92–98), moderate risk (82 to <92), and major risk (<82).

The PNI, originally proposed by Onodera et al. [6] in 1984, assesses serum albumin and total lymphocyte count to indicate both nutritional and immunological status, as follows: PNI = (10 × serum albumin [g/dL]) + (0.005 × total lymphocyte count [/mm^3^]). Lower PNI values indicate poorer nutritional and immunological status.

The CONUT score, introduced by Ignacio et al. [7] in 2005, evaluates nutritional status using a composite scoring system based on serum albumin, total cholesterol, and total lymphocyte count, with each parameter assigned a score according to predefined thresholds. Based on the total score, nutritional status is classified as no malnutrition (0–1), mild malnutrition (2–4), moderate malnutrition (5–7), or severe malnutrition (8–12).

### 2.4. Study Design and Analytical Strategy

#### 2.4.1. Association Between Nutritional Status and BMD

Correlation analyses were performed to examine the relationships between nutritional indices (GNRI, PNI, and CONUT) and BMD T-scores for the femoral neck and lumbar spine. Correlations among the nutritional indices were also evaluated.

#### 2.4.2. Association of Nutritional Status with BMD, Vitamin D, and Bone Turnover Markers

Patients were classified into well-nourished and malnourished groups according to each nutritional index. Malnutrition was defined as GNRI < 98, PNI < 40, and CONUT ≥ 2. Values above these thresholds were considered indicative of a well-nourished status. BMD, serum 25(OH)D levels, and bone turnover markers (total P1NP and TRACP-5b) were compared between the two groups to evaluate differences in clinical characteristics.

#### 2.4.3. Multivariate Analysis of Factors Associated with Bone Mineral Density

Multivariate analyses were conducted to determine whether nutritional status was independently associated with femoral neck BMD after adjusting for potential confounders.

### 2.5. Statistical Analysis

All statistical analyses were performed using EZR (Saitama Medical Center, Jichi Medical University, Saitama, Japan), a graphical user interface for R (The R Foundation for Statistical Computing, Vienna, Austria), which is a modified version of R Commander that incorporates statistical functions commonly used in biostatistical analyses. Group comparisons were performed using Student’s *t*-test or the Mann–Whitney U test, as appropriate, and correlations were assessed using Spearman’s rank correlation coefficient. Multiple linear regression analysis was performed with the femoral neck BMD T-score as the dependent variable because this outcome was continuous. Independent variables were selected based on clinical relevance and previous evidence regarding bone metabolism and included age, sex, BMI, and each nutritional index. All variables were simultaneously entered into the regression model. Multicollinearity was assessed using variance inflation factors. Because this was a retrospective observational study, an a priori sample size calculation was not performed. A post hoc power analysis using the final sample size of 108 patients showed adequate power to detect the observed correlation between GNRI and femoral neck BMD T-score (r = 0.48; power > 0.99) and moderate power to detect weaker correlations such as r = 0.26 (power = 0.782), at a two-sided alpha level of 0.05. A two-sided *p* value < 0.05 was considered statistically significant.

## 3. Results

### 3.1. Patient Characteristics

In total, 108 patients were included in the final analysis. Patient characteristics are summarized in Table 1. The mean age was 81.9 ± 8.8 years, and 37.0% of the patients were men. The mean GNRI and PNI were 93.4 ± 10.5 and 41.0 ± 5.5, respectively. The median CONUT score was 3 (range, 1–9). Nineteen (17.6%) patients received pre-fracture osteoporosis treatment, including active vitamin D monotherapy in 11, bisphosphonate monotherapy in 3, bisphosphonate plus active vitamin D in 2, denosumab in 1, selective estrogen receptor modulator (SERM) monotherapy in 1, and SERM plus active vitamin D in 1.

### 3.2. Association Between Nutritional Status and BMD

Correlation analyses were performed to evaluate the associations among nutritional indices and between nutritional indices and BMD T-scores at the femoral neck and lumbar spine. GNRI was significantly positively correlated with PNI (r = 0.703, *p* < 0.001). In contrast, GNRI and PNI were significantly negatively correlated with the CONUT score (r = −0.573 and r = −0.845, respectively; both *p* < 0.001).

**Figure 1:**
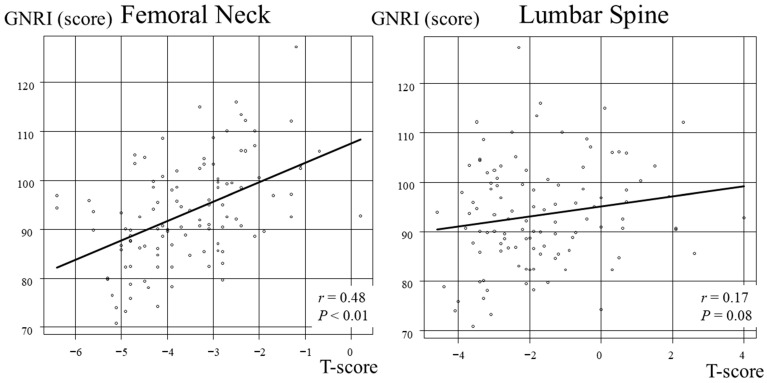
Correlation between the Geriatric Nutritional Risk Index (GNRI) and bone mineral density (BMD). GNRI showed a significant positive correlation with femoral neck BMD (r = 0.48, *p* < 0.01) but not with lumbar spine BMD (r = 0.17, *p* = 0.08).

**Figure 2:**
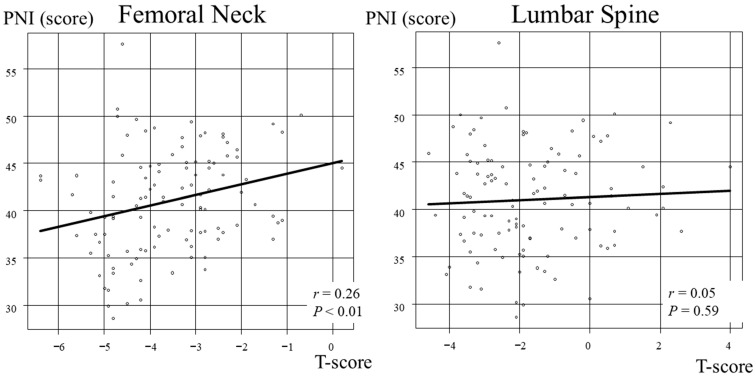
Correlation between the Prognostic Nutritional Index (PNI) and bone mineral density (BMD). PNI showed a significant, weakly positive correlation with femoral neck BMD (*r* = 0.26, *p* < 0.01) but showed no significant correlation with lumbar spine BMD (*r* = 0.05, *p* = 0.59).

**Figure 3:**
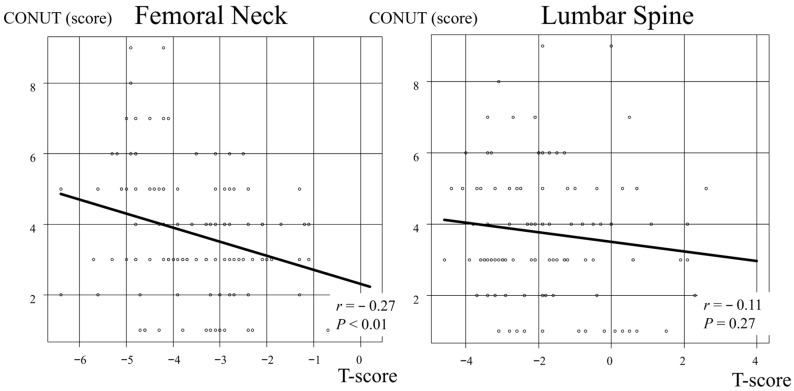
Correlation between the Controlling Nutritional Status (CONUT) score and bone mineral density (BMD). The CONUT score showed a significant negative correlation with femoral neck BMD (*r* = −0.27, *p* < 0.01) but not with lumbar spine BMD (*r* = −0.11, *p* = 0.27).

GNRI was significantly positively correlated with femoral neck BMD T-scores (r = 0.48, *p* < 0.01) and positively but non-significantly correlated with lumbar spine BMD T-scores (r = 0.17, *p* = 0.08) (Figure 1). Similarly, PNI was positively correlated with femoral neck BMD T-scores (r = 0.26, *p* < 0.01) but not with lumbar spine BMD T-scores (r = 0.05, *p* = 0.59) (Figure 2). In contrast, CONUT scores were negatively correlated with femoral neck BMD T-scores (r = −0.27, *p* < 0.01). However, no significant correlation was found between CONUT and lumbar spine BMD T-scores (r = −0.11, *p* = 0.27) (Figure 3).

### 3.3. Association of Nutritional Status with BMD, Vitamin D, and Bone Turnover Markers

Patients were classified into well-nourished and malnourished groups according to each nutritional index. Comparisons of BMD, serum 25(OH)D levels, and bone turnover markers between the well-nourished and malnourished groups based on the GNRI, PNI, and CONUT score are summarized in Table 2, Table 3 and Table 4, respectively.

Based on the GNRI, the malnourished group showed significantly lower femoral neck BMD and serum 25(OH)D levels and higher TRACP-5b levels than the well-nourished group. However, no significant difference was observed in lumbar spine BMD or total P1NP levels between the two groups.

Based on the PNI, the malnourished group had significantly lower femoral neck BMD and serum 25(OH)D levels and significantly higher total P1NP and TRACP-5b levels than the well-nourished group. However, lumbar spine BMD also did not differ significantly between the groups.

When classified by CONUT score, lumbar spine BMD was significantly lower in the malnourished group, whereas femoral neck BMD, serum 25(OH)D levels, total P1NP, and TRACP-5b levels did not differ significantly between the groups.

### 3.4. Multivariate Analysis of Factors Associated with BMD

Multiple linear regression analysis identified the factors independently associated with femoral neck BMD T-score. The results of the GNRI, PNI, and CONUT models are summarized in Table 5, Table 6 and Table 7, respectively. In the GNRI model, both the GNRI and sex were independently associated with femoral neck BMD T-score, whereas age and BMI were not significant predictors (Table 5). In the PNI model, the PNI, BMI, and sex were significantly associated with femoral neck BMD T-score (Table 6). In the CONUT score model, BMI, the CONUT score, and sex were independently associated with femoral neck BMD T-score (Table 7). No significant multicollinearity was observed among the independent variables in any model (all variance inflation factors <3).

## 4. Discussion

In the present study, we investigated the association between nutritional status—assessed using hematological indices—and BMD, serum 25(OH)D levels, and bone turnover markers in patients with proximal femoral fragility fractures. Poorer nutritional status was significantly associated with lower femoral neck BMD. Correlation analyses revealed that femoral neck BMD was positively correlated with GNRI and PNI, whereas it was negatively correlated with the CONUT score. When patients were classified by nutritional status, the malnourished groups exhibited lower femoral neck BMD and serum 25(OH)D levels, and higher bone turnover markers than the well-nourished groups, although these differences varied with the nutritional index used. Furthermore, multiple linear regression analysis revealed that nutritional indices remained independently associated with femoral neck BMD even after adjusting for age, sex, and BMI. Therefore, nutritional status may be closely related to bone metabolism and bone density in patients with proximal femoral fragility fractures.

Malnutrition has been increasingly recognized as an important clinical factor in orthopedic patients. Previous studies have mainly focused on the association between nutritional status and postoperative outcomes, including complications, functional recovery, and mortality [2,12,13,14]. However, relatively few studies have investigated the relationship between nutritional status and bone-related parameters, such as BMD, vitamin D levels, and bone turnover markers, particularly in patients with proximal femoral fragility fractures. Therefore, the present study focused on the association of hematological nutritional indices with BMD and bone metabolism markers, which were the primary outcomes of this study.

Several studies have investigated the association between nutritional status and BMD. In patients with type 2 diabetes mellitus (T2DM), GNRI was positively correlated with lumbar spine, total hip, and femoral neck BMD, and this association remained significant after adjustment for potential confounding factors. The findings indicate that GNRI-based nutritional status is independently associated with bone mass in this population [11]. Similarly, a large population-based study analyzing data from the National Health and Nutrition Examination Survey found a positive association between GNRI and femoral BMD in postmenopausal women, with higher GNRI values indicating a lower risk of osteoporosis [15]. Thus, nutritional status may be closely associated with bone density across various populations. Furthermore, studies have examined the relationships between GNRI, PNI, and the CONUT score and BMD. GNRI was identified as an independent protective factor against osteoporosis in older adults with T2DM, whereas PNI and CONUT scores showed weaker or no significant associations with osteoporosis after adjusting for confounding factors [16]. Overall, these studies suggest that nutritional status, particularly if GNRI-based, is associated with BMD and osteoporosis risk in several populations. However, evidence on the relationship between nutritional indices and bone parameters in orthopedic patients with fragility fractures remains limited. The present study established the association between nutritional indices and femoral neck BMD but not with lumbar spine BMD. This finding may be partly explained by degenerative changes in the lumbar spine, including osteophytes, vertebral sclerosis, and occult vertebral fractures, which may cause an overestimation of lumbar spine BMD as measured by DXA [17].

Previous studies have demonstrated the significance of nutritional status in bone metabolism. Malnutrition contributes to decreased bone mass and an increased risk of fragility fractures through multiple mechanisms, including insufficient protein and micronutrient intake, as well as chronic inflammation. Vitamin D is a key regulator of calcium homeostasis and skeletal metabolism, and deficiencies are highly prevalent in older adults. Poor nutritional status may be associated with lower serum 25(OH)D levels. A previous study in older adults found a significant positive correlation between serum 25(OH)D levels and the PNI; thus, individuals with better nutritional status tend to have higher vitamin D levels [18]. Besides vitamin D, nutritional status may influence bone turnover through systemic metabolic and inflammatory pathways. Chronic inflammation and inadequate nutrition can promote catabolic metabolism and increase bone resorption. Nutritional status markers, such as serum albumin, are associated with bone health and fracture risk in various clinical populations [19,20]. However, despite these findings, the relationship between commonly used hematological nutritional indices, such as the GNRI, PNI, and CONUT score, and bone metabolism markers remains insufficiently investigated, particularly in patients with fragility fractures. In the present study, GNRI- and PNI-based nutritional status was significantly associated with serum 25(OH)D levels and bone turnover markers. In particular, patients with poorer nutritional status tended to show higher levels of bone turnover markers, particularly P1NP and TRACP-5b, indicating increased bone turnover activity. Thus, malnutrition may be associated not only with reduced bone mass but also with abnormalities in bone metabolism in patients with fragility fractures.

In recent years, nutritional status has received increasing attention in orthopedic surgery due to its impact on perioperative complications. Although nutritional status also plays a role in osteoporosis pathophysiology, relatively few studies have examined the relationship between nutritional status and bone-related parameters in orthopedic patients, particularly those with fragility fractures. The present findings suggest that nutritional status is associated with BMD and bone metabolism markers in patients with fragility fractures. Therefore, osteoporosis management in patients with fragility fractures requires close monitoring of both BMD and nutritional status.

This study has several limitations. First, this was a single-center retrospective study with a relatively limited sample size, which may restrict the generalizability of the findings. Second, the study population exclusively comprised patients with proximal femoral fragility fractures, who are typically older and may have specific clinical characteristics. Therefore, the results may not be directly applicable to other orthopedic populations. Third, BMD and bone turnover markers were measured postoperatively and may have been influenced by the fracture itself, surgical stress, and the subsequent healing process. Furthermore, fracture-related inflammation and acute reactions can affect nutritional indices, thereby potentially influencing the assessment of nutritional status. Finally, seasonal variation in vitamin D levels may have affected serum 25(OH)D measurements but was not examined in the present study. Fourth, detailed information on comorbidities and medications other than pre-fracture osteoporosis treatment was not comprehensively available. Therefore, we could not fully account for the potential effects of diseases or medications that may influence bone metabolism, such as diabetes mellitus, rheumatoid arthritis, glucocorticoid use, vitamin K antagonist use, or antipsychotic drug use. In addition, some non-statistically significant findings should be interpreted with caution because the sample size was limited, the number of patients in some subgroups was small, and lumbar spine BMD may have been affected by degenerative changes or excluded vertebrae.

## 5. Conclusions

In this study, nutritional status assessed using hematological indices (GNRI, PNI, and CONUT score) was significantly associated with femoral neck bone mineral density and bone metabolism markers in patients with proximal femoral fragility fractures. In particular, poorer nutritional status was consistently associated with lower femoral neck BMD, lower serum 25(OH)D levels, and increased bone turnover activity. These findings suggest that nutritional status is closely linked not only to bone density but also to bone metabolic processes in this population. From a clinical perspective, the present results highlight the importance of comprehensive nutritional assessment in patients with fragility fractures, as it may provide additional information beyond conventional bone-related evaluations. Incorporating nutritional screening into routine clinical practice may contribute to improved risk stratification and optimization of osteoporosis management. Further prospective and multicenter studies are warranted to clarify the causal relationship between nutritional status and bone health and to determine whether nutritional interventions can improve bone density and clinical outcomes in this population.

## Figures and Tables

**Table 1:** Patient characteristics.

Age (years)	81.9 ± 8.8
Sex	Male, 40 (37.0%); female, 68 (63.0%)
BMI	20.7 ± 4.4
Diagnosis	Femoral neck fracture, 70 (64.8%); femoral trochanteric fracture, 38 (35.2%)
Surgical procedure	ORIF, 47 (43.5%); bipolar hemiarthroplasty, 61 (56.5%)
GNRI	93.4 ± 10.5
PNI	41.0 ± 5.5
CONUT score	3 (1–9)

Values are expressed as mean ± standard deviation, median (range), or number (%). BMI, body mass index; ORIF, open reduction and internal fixation; GNRI, Geriatric Nutritional Risk Index; PNI, Prognostic Nutritional Index; CONUT, Controlling Nutritional Status.

**Table 2:** Clinical parameters according to GNRI-defined nutritional status.

	Malnourished Group	Well-Nourished Group	*p* Value
*n*	72	36	
BMD (femoral neck)	−3.9 ± 1.2	−2.9 ± 1.0	<0.01
BMD (lumbar spine)	−1.7 ± 1.7	−1.6 ± 1.7	0.68
25(OH)D (ng/mL)	9.2 ± 5.3	12.0 ± 7.0	0.03
Total P1NP (ng/mL)	158.1 ± 145.0	117.5 ± 83.6	0.13
TRACP-5b (mU/dL)	544.8 ± 290.3	415.2 ± 150.2	0.01

Values are expressed as mean ± standard deviation. GNRI, Geriatric Nutritional Risk Index; BMD, bone mineral density; 25(OH)D, 25-hydroxyvitamin D; Total P1NP, total procollagen type I N-terminal propeptide; TRACP-5b, tartrate-resistant acid phosphatase 5b.

**Table 3:** Clinical parameters according to PNI-defined nutritional status.

	Malnourished Group	Well-Nourished Group	*p* Value
*n*	47	61	
BMD (femoral neck)	−3.8 ± 1.2	−3.4 ± 1.3	0.04
BMD (lumbar spine)	−1.9 ± 1.6	−1.5 ± 1.8	0.27
25(OH)D (ng/mL)	8.5 ± 3.9	11.4 ± 7.0	0.01
Total P1NP (ng/mL)	176.0 ± 136.4	120.4 ± 117.8	0.03
TRACP-5b (mU/dL)	559.4 ± 289.6	457.1 ± 224.1	0.04

Values are expressed as mean ± standard deviation. PNI, Prognostic Nutritional Index; BMD, bone mineral density; 25(OH)D, 25-hydroxyvitamin D; Total P1NP, total procollagen type I N-terminal propeptide; TRACP-5b, tartrate-resistant acid phosphatase 5b.

**Table 4:** Clinical parameters according to CONUT score-defined nutritional status.

	Malnourished Group	Well-Nourished Group	*p* Value
*n*	95	13	
BMD (femoral neck)	−3.6 ± 1.3	−3.3 ± 1.1	0.43
BMD (lumbar spine)	−1.8 ± 1.7	−0.9 ± 1.5	0.04
25(OH)D (ng/mL)	9.6 ± 4.5	14.1 ± 11.8	0.53
Total P1NP (ng/mL)	149.5 ± 132.3	108.2 ± 96.4	0.24
TRACP-5b (mU/dL)	514.0 ± 266.2	441.2 ± 182.1	0.25

Values are expressed as mean ± standard deviation. CONUT, Controlling Nutritional Status; BMD, bone mineral density; 25(OH)D, 25-hydroxyvitamin D; Total P1NP, total procollagen type I N-terminal propeptide; TRACP-5b, tartrate-resistant acid phosphatase 5b.

**Table 5:** Multivariate linear regression analysis of factors associated with femoral neck BMD T-score (GNRI Model).

Variable	B	95% CI	*p* Value	VIF
Age	−0.0167	−0.039 to 0.006	0.142	1.12
Sex (male vs. female)	1.063	0.671–1.455	<0.001	1.05
BMI	0.0289	−0.038 to 0.095	0.390	2.46
GNRI	0.0466	0.018–0.075	0.002	2.58
Adjusted R^2^ = 0.412				

BMD, bone mineral density; BMI, body mass index; GNRI, Geriatric Nutritional Risk Index; CI, confidence interval; VIF, variance inflation factor. B indicates the non-standardized regression coefficient.

**Table 6:** Multivariate linear regression analysis of factors associated with femoral neck BMD T-score (PNI Model).

Variable	B	95% CI	*p* Value	VIF
Age	−0.0198	−0.042 to 0.003	0.082	1.10
Sex (male vs. female)	1.009	0.614–1.404	<0.001	1.03
BMI	0.108	0.065–0.152	<0.001	1.04
PNI	0.0478	0.013–0.083	0.008	1.04
Adjusted R^2^ = 0.395				

BMD, bone mineral density; BMI, body mass index; PNI, Prognostic Nutritional Index; CI, confidence interval; VIF, variance inflation factor. B, the unstandardized regression coefficient.

**Table 7:** Multivariate linear regression analysis of factors associated with femoral neck BMD T-score (CONUT score Model).

Variable	B	95% CI	*p* Value	VIF
Age	−0.0197	−0.042 to 0.003	0.083	1.10
Sex (male vs. female)	0.989	0.595–1.384	<0.001	1.03
BMI	0.108	0.064–0.152	<0.001	1.04
CONUT score	−0.146	−0.252 to −0.040	0.007	1.04
Adjusted R^2^ = 0.396				

BMD, bone mineral density; BMI, body mass index; CONUT, Controlling Nutritional Status; CI, confidence interval; VIF, variance inflation factor. B, the unstandardized regression coefficient.

## Data Availability

The data that support the findings of this study are available from the corresponding author upon reasonable request.

## Abbreviations

The following abbreviations are used in this manuscript:

BMD	Bone mineral density
BMI	Body mass index
DXA	Dual-energy X-ray absorptiometry
GNRI	Geriatric Nutritional Risk Index
PNI	Prognostic Nutritional Index
SERM	Selective estrogen receptor modulator
